# Supplementary material for: Causal Relationship between Aging and Anorexia Nervosa: A White-Matter-Microstructure-Mediated Mendelian Randomization Analysis
Source: Biomedicines. 2024 Aug 16;12(8):1874. doi: 10.3390/biomedicines12081874 (PMC11351342; doi:10.3390/biomedicines12081874)
Supplement: Supplementary file 1 [file biomedicines-12-01874-s001.zip › Supplementary Figure/Figure S4/Singe SNP plot of MR analysis for TL to ubm-b-1679.pdf]

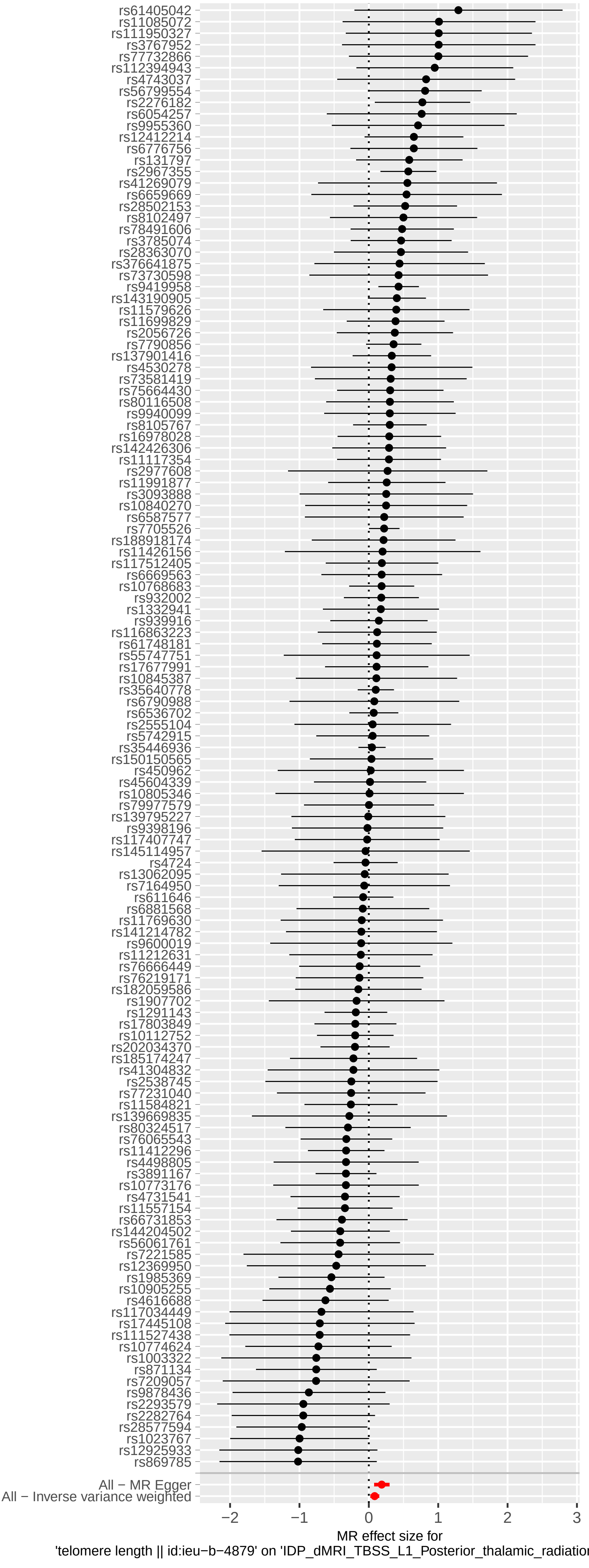
rs61405042
rs11085072
rs111950327
rs3767952
rs77732866
rs112394943
rs4743037
rs56799554
rs2276182
rs6054257
rs9955360
rs12412214
rs6776756
rs131797
rs2967355
rs41269079
rs6659669
rs28502153
rs8102497
rs78491606
rs3785074
rs28363070
rs376641875
rs73730598
rs9419958
rs143190905
rs11579626
rs11699829
rs2056726
rs7790856
rs137901416
rs4530278
rs73581419
rs75664430
rs80116508
rs9940099
rs8105767
rs16978028
rs142426306
rs11117354
rs2977608
rs11991877
rs3093888
rs10840270
rs6587577
rs7705526
rs188918174
rs11426156
rs117512405
rs6669563
rs10768683
rs932002
rs1332941
rs939916
rs116863223
rs61748181
rs55747751
rs17677991
rs10845387
rs35640778
rs6790988
rs6536702
rs2555104
rs5742915
rs35446936
rs150150565
rs450962
rs45604339
rs10805346
rs79977579
rs139795227
rs9398196
rs117407747
rs145114957
rs4724
rs13062095
rs7164950
rs611646
rs6881568
rs11769630
rs141214782
rs9600019
rs11212631
rs76666449
rs76219171
rs182059586
rs1907702
rs1291143
rs17803849
rs10112752
rs202034370
rs185174247
rs41304832
rs2538745
rs77231040
rs11584821
rs139669835
rs80324517
rs76065543
rs11412296
rs4498805
rs3891167
rs10773176
rs4731541
rs11557154
rs66731853
rs144204502
rs56061761
rs7221585
rs12369950
rs1985369
rs10905255
rs4616688
rs117034449
rs17445108
rs111527438
rs10774624
rs1003322
rs871134
rs7209057
rs9878436
rs2293579
rs2282764
rs28577594
rs1023767
rs12925933
rs869785
All − MR Egger
All − Inverse variance weighted
−2
−1
0
1
2
3
MR effect size for
'telomere length || id:ieu−b−4879' on 'IDP_dMRI_TBSS_L1_Posterior_thalamic_radiatio
